# Dissertation on the Diseases of the Maxillary Sinus

**Published:** 1843-03

**Authors:** Chapin A. Harris

**Affiliations:** Professor of Practical Dentistry in the Baltimore College of Dental Surgery, &c.


THE AMERICAN

### JOURNAL OF DENTAL SCIENCE.

VOL. III.] · MARCH, 1843. [No. 3.

### ARTICLE I.

*Dissertation on the Diseases of the Maxillary Sinus.* Read before the American Society of Dental Surgeons, at their third annual meeting; held in the city of Boston, July 20th, 1842. By CHAPIN A. HARRIS, M. D., D. D. S., Professor of Practical Dentistry in the Baltimore College of Dental Surgery, &c.

(Concluded from page 132.)

### OF TUMOURS OF ITS LINING MEMBRANE AND PERIOSTEUM.

THE lining membrane and periosteal tissue of the maxillary sinus occasionally become the seat of fungous and other descriptions of tumours, and in consequence of the concealed situation of this cavity, morbid productions originating in it, often, as has been previously remarked, make considerable progress before they attract attention; and hence, the efforts of art for their cure, which might otherwise frequently be successful, in most instances prove unavailing. The presence of a tumour in this cavity may give rise to all the diseases to which its osseous walls are liable, as well also as to most of those that are incident to its soft tissues. As soon as a morbid growth here, has filled the sinus, it, as it continues to augment in size, presses upon the lining membrane, and excites in it inflammation and sometimes ulceration, and causes its secretions to become vitiated. A diseased action is communicated to the periosteum of the surrounding osseous walls; it ceases to furnish them with the healthy juices which they require for their preservation, thickens, ulcerates, and is at once destroyed, or exudes a corrosive fluid. The bony parietes of the sinus are soon softened or become affected with caries or necrosis, and one or more fistulous openings are formed through the cheek, alveoli, or palatine arch.

But these are not the only effects that result from tumours situated in this cavity. As they increase in volume, after having filled the sinus, they gradually distend and displace its bony parietes; the floor of the orbit is sometimes elevated, and the eye more or less forced from its socket; the palatine arch and alveolar ridge are depressed, the teeth loosened and caused to drop out, and when the tumour is of a soft fungous nature, it not unfrequently escapes through the alveoli into the mouth, and after forcing the jaws asunder to their greatest extent, protrudes from it in enormous masses. Bertrandi gives the history of a case of polypus excrescence of the antrum, which, after having destroyed the palate, anterior part of the maxillary bone, and filled the mouth, forced itself up into the orbit, elevated its roof, pressed upon the brain, and ultimately occasioned apoplexy and death.* Other similar cases are on record. Mr. Cooper says there are three specimens of diseased antrum in the museum of London University College. The tumour in two of these, had "made its way from the antrum to the brain." The third was taken from a patient of his, which had died. The tumour in this case, which was of a medullary and scirrhous character, forced itself up into the orbit, displaced the eye, and ultimately caused the death of the patient. The same author mentions another case, the subject of which was a boy in St. Bartholomew's Hospital, who had a tumour of the antrum which "made its way through the orbiter plate of the frontal bone and cribriform plate of the ethmoid into the cranium," and though the portion of it that entered the brain was as large as a small orange, he says the boy was only in a comatose state about forty-eight hours previously to his death.†

Tumours occupying the maxillary sinus do not always originate in its lining membrane or periosteum. They sometimes arise from the pituitary membrane of the nose, frontal sinus, or ethmoidal cells, and after having found their way into this cavity,

* Vide, Traite de ses Operations Chirurgicales, p. 369.

† Vide Professor Reese's Appendix to Cooper's Surgical Dictionary, American edition of 1842, page 29.

augment in size, until they produce the effects just described. Some suppose that the morbid productions found here, originate more frequently in the cells of the ethmoid bone, than in the lining membrane of this cavity;* but I am disposed to believe that this opinion is not well founded, and that it has chiefly resulted from the great liability of most kinds of tumours of the maxillary sinus, to be reproduced after having been extirpated,—which is often attributable to the continuance of the cause that gave rise to them in the first instance, or to their imperfect removal. That they do however sometimes originate in the ethmoidal cells, there can be no question.

It sometimes happens that tumours having their seat in the antrum, after having filled it, make their way into the nose, where they acquire a size equal to, or even greater than that to which they had previously attained here, thus dividing themselves, as it were, into two parts—one occupying this, and the other, one of the nasal cavities. Occurrences of this sort are not unfrequent, and they sometimes lead to the adoption of an incorrect opinion, with regard to the real seat of the disease. Thus, a polypus of the antrum is occasionally mistaken for one of the nose, and the error frequently not discovered, until an attempt is made to remove it.

The character of morbid growths in this cavity is exceedingly variable, as much so as is the state of the constitutional health of different individuals, and the causes that give rise to them. They not only vary in their appearance and structure, but they vary in their malignancy. Some are of a healthy flesh colour, soft, sensible, but not painful, and present a smooth, regular surface; others varying in their consistence from hard to soft, and in their colour from a pale yellow to a deep red or purple, present a rough, irregular, and not unfrequently ulcerated surface, and are more or less sensitive to the touch and painful. Some have their origin in the mucous membrane, and others, both in this, and the periosteum. Some are attached by a broad base, and others, it is said, are connected only by a mere peduncle.

But as it regards this latter description of tumours, which are usually designated by the name of polypi, their occurrence in the

* Vide Traite des Maladies de la Bouch, t. i. p. 210, &c.

maxillary sinus is questioned by some writers. Sir Benjamin Brodie does not believe that they ever form in this cavity;* and in this opinion Mr. S. Cooper fully concurs;† but that they are occasionally met with here, seems nevertheless to be pretty conclusively established. A case described by M. Bertrandi in his treatise on Operative Surgery, page 369, has already been referred to, and Bordenave, in his observations on the diseases of the antrum maxillare, gives the history of a case treated by M. Doublet.‡ Rusch declares that he has twice seen polypus of this cavity, and Petitt, Levrette and other writers also affirm that they have witnessed polypi here.‖ The occurrence then of polypi in the maxillary sinus, although very rare, it must be admitted does sometimes happen. But other descriptions of tumours are certainly more frequently met with in this cavity. Of these, some are of a simple fibrous, sarcomatous, or osteo-sarcomatous nature,§ and when thoroughly extirpated, are seldom reproduced; others are of a medullary, cancerous, or carcinomatous character. These last, although originating in the mucous membrane, if long neglected, are very liable to be reproduced after their removal, and generally occasion the death of the patient.

It sometimes happens that several fungi, and from opposite and various points in this cavity, spring up. The chances of cure, when this is the case, especially if they are of a malignant character, are greatly lessened.

Tumours in the maxillary sinus seldom grow very fast during the early stages of their formation; but, as they enlarge, the neighbouring parts become involved in the diseased action, and consequently furnish them with fluids less healthy in their qualities, and thus cause them to assume a character of greater malignancy, and generally to increase more rapidly in size.

* Vide London Medical Gazette, for December, 1834. p. 850.

† Vide Professor Reese's Appendix to Cooper's Surgical Dictionary, American edition, 1842, p. 29.

‡ Vide Mem. de l'Academie Royale de Chirurg. 12mo. t. 13, p. 393.

‖ Vide Traite des Maladies de la Bouch, tom. 1, p. 212, and sur cure des Polypes de la matrice, de la gorge, et du nez, page 253.

§ Vide Professor Reese's Appendix to Cooper's Surgical Dictionary, American edition, 1842.

Having premised these few general observations on tumours of the maxillary sinus, I shall proceed to describe the principal signs by which their existence here is indicated.

*Symptoms.*—The occurrence of tumours in the maxillary sinus is rarely accompanied, previously to their having obtained a size sufficiently large to fill it, by symptoms differing materially from those occasioned by many of the other affections that locate themselves here. But, after they have filled the sinus, the indica-cations soon become less equivocal. Swelling of the cheek, depression of the palatine arch and alveolar ridge, loosening of the superior molar teeth of the affected side, inflammation and sponginess of the gums, elevation of the floor of the orbit, and protrusion or concealment of the eye, are symptoms which result from the presence of tumours in this cavity, but they are not peculiar to these affections alone; they are also produced by mucous engorgement of the sinus. But when to these is superadded the discharge of a bloody sanies from the nose, or from one or more fistulous openings through the cheek, alveolar ridge, or palatine arch, the diagnosis will be conclusive; and the existence of a tumour in the antrum will then be established beyond doubt.

There are also other signs by which the occurrence of a morbid growth in this cavity may be known; as for example,—the dropping out of the superior molares of the affected side, and the protrusion of portions of the tumour through the alveoli.

The pain is seldom severe until the tumour has filled the cavity, except it be from its inception of a malignant character; but as it augments in size and forces the walls of the sinus asunder, it becomes more and more severe. It sometimes, during the progress of the disease, becomes almost excruciating. In a case of fungus hæmatodes of this cavity, which the author, a few years since, had an opportunity of witnessing, the patient was in the habit of taking upwards of two tea-spoonsful of black drop at a time, for the procurement of ease and sleep.

But in addition to the foregoing symptoms, several of the affections already treated on, together with all the effects produced by them, not unfrequently result from tumours in this cavity. Inflammation and ulceration of its lining membrane, a purulent condition of its secretions, and caries, necrosis and a softening of its osseous walls, seldom fail to follow some of the stages of the formation of the morbid productions now under consideration.

The symptoms peculiar to each variety of tumour, it is not necessary to mention, inasmuch as they are given by most writers on general surgery.

*Causes.*—Most writers on the affections of the maxillary sinus, are of the opinion that tumours in this cavity result spontaneously, as a consequence of some specific constitutional vice, independently of local causes. I do not however believe that they are ever thus originated. But that a bad habit of body or some constitutional vice is necessary to the production of the affections under consideration, I do not doubt, but that this is capable of giving rise to them in parts uninfluenced by local irritation, I think exceedingly questionable. Having however already expressed my views with regard to the agency exerted by particular habits of body and constitutional vices in the production of diseases in this cavity, it will not be necessary to repeat what I have before said upon the subject.* It will be sufficient to remark that most, if not all of the morbid excrescences here met with, result from local irritation and constitutional vices; and that both are necessary to their production.

Scorbutic and scrofulous habits, and persons whose general health has been impaired by certain constitutional diseases,—such as the venereal, protracted inflammatory and bilious fevers, dyspepsia, &c., are most subject to tumours of the maxillary sinus; but every thing in fact, which has a tendency to increase the irritabilty of the soft tissues of the body may be considered as so many predisposing causes. The local causes are the same as those of most of the other morbid affections of this cavity. Diseased teeth, gums and alveolar process are probably among the most common. The irritation produced by these so frequently extends itself to the antrum, that their agency in the production of tumours here, cannot be questioned. There are, however, other causes of irritation to which this cavity is exposed, such for example, as blows upon the cheek, wounds, &c.

*Treatment.*—It is only in the early stages of the formation of tumours in the maxillary sinus, that surgical treatment can be

* Vide introductory remarks, pp. 27—9.

adopted with success, and even then, their entire extirpation is necessary. If this be not accomplished, a speedy return of the disease may be expected. But, preparatory to the removal of the diseased structure, a large opening should be made into the antrum, so as to expose as much of it as possible; and with regard to the most proper place for effecting this, Deschamps, recommends when the alveolar ridge has been started, the removal of the first or second molaris, and the perforation of the sinus through its socket with a "three-sided trocar of suitable dimensions." But when the alveolar ridge and teeth are sound, he directs the opening to be made through the outer wall of the sinus above the ridge, and this he thinks, on account of its being more direct, is preferable to the other mode.* An opening may be easily effected in either way into the sinus, as its walls are generally, so much softened as to offer but little resistance.

When the opening is to be made through the external parietes, the instrument recommended by Mr. Thos. Bell,† to be employed for cutting away the bone after it has been exposed, is "a strong hooked knife," which is probably as well adapted to the purpose as any that could be used. Some surgeons employ strong curved scissors, but the hooked knife, I should think preferable.

A free opening having been effected into the antrum, a finger of the operator should be introduced, and the nature of the diseased structure ascertained. This done, he will be enabled to determine on the procedure to be had recourse to for its removal. If the tumour partakes of the character of those called polypi, it may be seized with a pair of forceps and torn away, but if it be attached by a broad base, its extirpation will be most readily effected with a knife. But, even with this, it is often exceedingly difficult to effect its total removal, so that it not unfrequently becomes necessary to employ the actual cautery; for, if any small portions be left behind, as has before been stated, a reproduction of the disease, will generally very soon take place. When the disease has originated, or is seated, in the periosteum, the cautery has proved to be the most effectual means of preventing its return of any that has been tried. The French surgeons have applied

* Malades des Fosses Nazales, sec. ii. art. iv. p. 244.

† Anat. Phys. and Diseases of the Teeth, p. 282.

it with great success. Desault, in a case of fungous tumour, succeeded in effecting a cure after three applications. The root of the disease, by the employment of this, can often be destroyed, when less effectual means would fail. But it is important when it is had recourse to, that it should have such a degree of heat, as to accomplish this object instantaneously, else, the inflammation that would otherwise be excited by its application in the surrounding parts, would greatly retard, if it did not prevent the cure. The remarks of Mr. Thos. Bell upon this subject, who says, "the white heat should be employed,"* are worthy of attention.

In remarking upon the bold practice of the French surgeons in the treatment of these affections, the author just quoted says, it "Is worthy of our praise and imitation;" and, continues he, "the timidity which, until very lately, almost excluded the use of the actual cautery in this country, has been one cause, and that a very prevalent one, of failure in the treatment of some of these cases; but it is not so easy to account for the still more culpable dread, which has in so many instances prevented any attempt from being made to extirpate the disease; a degree of pusillanimity which is at once an opprobrium on the profession, and a fatal injustice to the sufferers, who thus abandoned to the unrestrained progress of the disease, are left to perish by a lingering and most painful process, without even an attempt being hazarded for their relief."

The foregoing comparison, instituted by Mr. Bell, between the practice of the French and English surgeons in the treatment of tumours of the maxillary sinus, is correct; but it is due to truth to say, that the bold practice of the former has been fully and successfully emulated by American surgeons. Dr. A. H. Stevens, Professor of Surgery in the University of New York, in 1823, in a case of fungus tumour, attached by a broad base to the lower part of the antrum, removed a large portion of the lower and anterior parts of the upper jaw. The patient recovered and is said to be living at the present time.† In 1841, Dr. J. C. Warren, of Boston, for a case of cephalomatous tumour of this cavity, re-

* Anat. Phys. and Diseases of the Teeth, p. 282.

† Appendix to Cooper's Surgical Dictionary, p. 30.

moved the superior maxillary bone. This operation also, was successful.* The same operation was performed soon after, and for the removal of a tumour of the antrum, with success, by R. D. Mussey, of Cincinnati, Ohio.†

But the operation for the removal of the superior maxillary, did not originate with American surgeons; Velpeau says it was performed by Acoluthus in 1693, for a tumour of the face.‡ By a reference however, to the history of the case as given in a memoir of the Academy of the Curiosities of Nature,|| it will be perceived that the tumour originated in the maxillary sinus, and that only a part of the jaw-bone was removed. If however, we can believe Wiseman, this most formidable operation was performed at a still earlier period. He says in his surgery, the first edition of which was published in 1676, "That he cut into a man's cheek, sawed in pieces the alveolus, and took out the whole jaw, and cured him."§ But although the operation may have been performed thus early, it does not at all detract from the credit due to modern surgeons, since the method of effecting it, is at least, original with them.

Thus it is perceived, that the diseases under consideration not unfrequently call for one of the most formidable operations in surgery, and that by it, many unfortunate sufferers have been snatched, as it were, from the very jaws of death. But notwithstanding the performance of even this operation, the application of the cautery often becomes necessary to prevent a reproduction of the excrescence, and there are many cases in which it cannot be repressed by this. The result of the most thorough and best directed treatment depends on the state of the constitutional health and the nature of the disease. In depraved habits and shattered constitutions, if the tumour be of a carcinamatous character, a cure need never be expected.

The hemorrhage, during the operation for the removal of tumours of the antrum, is sometimes so profuse as to require very prompt and active means to arrest it. It may generally, however, be controlled by the employment of compresses and suitable styp-

* Boston Medical and Surgical Journal for 1842.

† Western Lancet for 1842.

‡ Velpeau's Operative Surgery, p. 263.

|| Decad 3, ann. 4o. Obs. 57.

§ Vide Wiseman's Surgery, page 285.

tics, but should these fail, the actual cautery should be had recourse to.

The history of the following cases, promiscuously taken from various works, will perhaps furnish a more correct idea of the methods of treatment most proper to be pursued, than any description which could otherwise be given. The first four cases are taken from the Memoirs de l'Academie Royale de Chirurgie.*

CASE XXIII.—A man about thirty-five years of age, had a fleshy tumour about the size of a large pea, situated in a space formed by the decay of the first and second superior molares of the left side. This tumour caused a dull pain; it was excised, and the actual cautery applied to arrest the bleeding and destroy remaining portions of the excrescence. It re-appeared, and three months after was double the size of the former, and impeded mastication. The two decayed teeth were loose and the others were painful; and a fetid matter escaped through the nose and mouth.

After the extraction of the two decayed teeth, M. Dubertrand, discovering that the tumour had its seat in the antrum, seized it with polypi forceps and brought the whole of it away. After the extraction of the tumour, the opening through the alveolus was large enough to admit the little finger. M. Dubertrand next destroyed such portions of the alveoli and maxillary bone as were decayed. But after the extirpation of the tumour, he found it necessary to introduce a plug of cotton into the antrum, to arrest the hemorrhage that followed the operation.

The secretions of the maxillary sinus ceased to exhale an unpleasant odour, in three days they became healthy, and in less than one month, the patient was restored to health, and the opening from the mouth into this cavity was closed with firm granulations.

The tumour just described was of the simplest kind, but had it not been completely eradicated, it would doubtless have soon reappeared.

CASE XXIV.—Acoluthus, reports the case of a woman thirty years of age, who in 1693, came to Pologne in Silesia, in search of aid for a peculiar disease of the antrum, under which she was labouring. Some time after the extraction of a tooth from the

* Tome 13, obs. 1, 5 and 7th, pages 372, 387 and 424.

left side of the upper jaw, a small tumour appeared in its alveolus, and it made such progress, that in two years, it attained the size of a double fist. It occupied nearly the whole cavity of the mouth, and distended the jaw to such a degree that it was feared it would rupture it. The lower jaw was depressed, the lips could not be made to meet, and the tumour increased so fast, that in a few weeks, the woman's life was despaired of—she being threatened with suffocation, hunger and thirst. Under these circumstances, Acoluthus determined to attempt a cure.

The tumour was very hard and occupied the greatest part of the palatine arch; the upper teeth of the left side were in its centre. The operation was commenced by enlarging the mouth, beginning at the commissure of the lips, and passing it transversely through the cheek. This enabled Acoluthus to attack the exterior of the tumour with a curved bistoury. The excrescence was as hard as cartilage and scarcely yielded to cutting instruments applied by a strong hand. He however succeeded in bringing three or four teeth together with a portion of the superior maxillary bone. But the operation as yet had extended only to the exterior half of the tumour; the other which filled the palatine fossæ, he says, it was impossible to bring away. The removal of that was effected only by piece-meal and at different times. The operation was long, laborious and very painful. The actual cautery was applied to the bleeding vessels and fungous flesh. The appearance of the patient, a few days after the operation was such as to inspire hope for a favourable termination of the disease. The actual cautery was applied several times, and finally there were no indications of a reappearance of the excrescence, except at the point where it had first originated. Some portions of bone, were afterwards found to be carious, and the removal of these was followed by a prompt and speedy cure.

This was the operation alluded to by Velpeau, as embracing the removal of the superior maxillary, previously noticed, but from the description here given of it, it would appear that only a small portion of the bone was taken away. The alveolar ridge and anterior parietes of the sinus was all that was removed. The history of the case, however imperfect as it is, and the result of its treatment, proves that the resources of art are adequate to the cure of many of the most formidable and threatening of the affections of this cavity, if their employment be not delayed too long.

CASE XXV.—In 1755, M. Doublet was consulted by a lady thirty-nine years of age, who had a tumour the size of a hazlenut at the internal angle of the left eye, upon the *os unguis.* This was movable and hard, but the patient experienced no pain from it. Some time after she complained of difficult respiration, through the left nasal cavity. Upon examination, M. Doublet discovered a polypus of a soft flabby consistency. This he thought should be extracted before attempting to remove the exterior tumour, which, having effected, that subsided and the patient enjoyed good health for four months. At the expiration of this time, a tumour three-fourths larger than the first made its appearance, which was extracted, and the patient again remained well for one month. But by this time, another polypus had made its appearance in the left nostril, softer than the first. This was treated by escharotics, and some time after a fourth polypus made its appearance, and a fourth tumour showed itself at the angle of the eye. The progress of these was more rapid than any which had preceded them, and they were accompanied by violent headache. The patient's general health, notwithstanding the local affection, was good, and the tumour and polypus did not exhale a fetid odour. The glands of the mouth were swollen. Alteratives were prescribed and an issue was opened. These appeared to give the patient some relief, but the tumour at the angle of the eye notwithstanding made such progress, and the maxillary sinus, which before was not suspected of being diseased, became so completely filled, that the palatine process of the maxillary bone, separated. This was followed by a considerable swelling of all the internal membrane of the mouth, the gums became hard and black, and in the month of March, 1758, the patient died.

On submitting the diseased part to a post mortem examination, a large tumour, says M. Doublet, was found in the antrum, which completely filled it, and it had attained double this size under the zygomatic arch and near the angle of the eye. The palatine process of the maxillary bone was pressed out about the width of two fingers; the upper row of teeth were displaced, and yet there was neither ulcer in the soft parts nor caries in the hard. The two tumours were scirrhous and no appearance of polypus was observed in the nose.

What the result of proper surgical treatment would have been in this case, had the seat of the disease been known, it is impossible to conjecture, since not a word is said concerning the causes, neither local nor constitutional, that gave rise to the tumour. There is nothing, therefore, in the history of the case, of special interest to the practitioner, and I have given place to it here only for the purpose of showing the importance of ascertaining the seat of morbid productions which develop themselves in the nose. It is not at all improbable that, had it been known in the case just described, a more successful plan of treatment might have been instituted, and the life of the patient saved.

CASE XXVI.—A young lady of Picardy having been exposed to the changes of weather for three years, in attending to business which required of her to be much on horseback, experienced, at the end of the first year, a chilly sensation in her left cheek; this increased, and her cheek became swollen, and her molar teeth of the affected side loosened and two dropped out.

The swelling of her cheek increased, and she was affected with lancinating pains in that side of her face; her breath became offensive and she lost two more teeth. Becoming alarmed, she went to Rouen to obtain medical advice, but receiving no satisfaction, she afterwards went to Paris, and applied, November 20th, 1740, to M. Croissant de Garengeot, who found her face greatly disfigured. Her mouth, he says, was on the right side, the left side of her nose much elevated, the left cheek very large, and the upper lip of the same side greatly thickened. Bluish flesh of the size of an olive occupied the alveoli of the teeth which had dropped out, the left side of the roof of the palate was thrown inwards and resembled the exterior projection of the cheek. The anterior wall of the antrum had become softened as well also as that of the left nasal bone, and the whole cavity was filled with fungous flesh.

M Garengeot commenced the operation by seizing the bluish excrescence which had appeared through the alveoli with a hook and cutting it away; and he says he incised transversely, every day, from within the mouth, the buccinator muscle, and brought away part of it as well as the flesh which so much augmented the size of the jaw.

The hemorrhage was so abundant that it was impossible to proceed further with the operation. The excrescence was rapidly reproduced after each operation; these excisions were repeated seven or eight times in six weeks, and the hemorrhage, each time, was very great. The seat of the disease was in the anterior of the sinus. The fungous flesh contained in this cavity was removed, as well also as some osseous projections.

The excrescence continuing to be reproduced, the consent of the patient, who had until now refused to have the actual cautery applied, having been obtained, its use was resorted to, twice a day, for eight days. The success, says M. Garengeot, which followed this treatment was incredible. The flesh soon took on a healthy consistency, the palatine arch returned about two-thirds to its natural situation, and the bad odour of the mouth gradually disappeared.

The application of the cautery was continued, once a day, for three weeks, and the patient did nothing more than to use a slightly stimulating and astringent gargle. On the 20th of March she returned home cured.

It is very probable that had the operation in the case just described been thorough, there would have been no return of the disease, for it is evident from the description which M. Garengeot gives of the operation, that the seat of the affection was not reached until it had been repeated seven or eight times; and then, I think it very likely, not until he had recourse to the actual cautery.

The utility of the actual cautery, not only for the purpose of thoroughly destroying every remaining vestige of fungous tumours of the antrum maxillare after their removal, but also for the suppression of hemorrhage, would seem to be fully established by the result of the treatment of cases twenty-four and six. That there are cases where it will fail to prevent their reproduction there is no question, but this does not detract from its value.

The employment of arsenical preparations has, in some instances, been found highly advantageous in repressing the growth of fungous excrescences. The following case is cited by Mr. Thomas Bell as an example.*

* Anat. Phys. and Diseases of the Teeth, p. 283.

CASE XXVII.—"James Woodly was admitted into Guy's Hospital, September 4th, 1821, for a fungous exostosis, which arose from the antrum maxillare, and made its way through the palate. After his admission he had the fungus removed two or three times, and a variety of caustic applications were afterwards made use of; notwithstanding which the tumour reappeared. At length Sir A. Cooper, after having made an incision from the corner of the mouth outwards through the cheek, removed the tumour from a greater depth than had previously been effected. After this operation the wound in the cheek readily healed, and the following strong solution of arsenic was daily applied to the part from whence the tumour had been removed.

℞. Arsenic, oxyd. alb. ʒ vi.
Potass. Subcarb. q. s.
Aq. Distillat. M. ft. solutio.

The solution required to be diluted in the first instance on account of its occasioning him a good deal of pain, in a few days, however, he used it of the strength mentioned in the formula. It was applied regularly every afternoon, after which he did not take any food until the following day. At the time of its application he had a piece of oiled silk, of a horse-shoe shape, passed into the mouth, its sides being turned up to prevent the solution escaping into the mouth: his head then hanging down over a basin, a piece of sponge moderately saturated with the solution was applied to the disease upon the oiled silk, pressed against the part; such of the solution as was then pressed out, passed along the channel of the oiled silk into the basin over which the head was hanging, and the salivia escaped behind the oiled silk into the same utensil. He kept the sponge in this situation until it gave him considerable pain, when it was removed and the mouth carefully washed. He suffered great pain in his mouth during the period of the cure; but the arsenic did not produce any other unpleasant symptoms. This application was continued for a few weeks, at the end of which time he was completely cured; a cavity being left in the site of the tumour, which however, gradually became covered by a continuation of the membrane which naturally lines the palate."

There are a number of highly interesting cases of sarcomatous, carcinamatous, and other kinds of tumours of the maxillary sinus, in Jourdain's Treatise on the Surgical Diseases of the Mouth; some of which, I had intended to introduce into this treatise, but apprehending that it would extend it to too great a length were I to do so, I have concluded to omit their insertion. A number of other equally interesting cases reported in various other works,* are for the same reason excluded.

But before dismissing this branch of the subject, I will add the history of one more case of tumour of this cavity, taken from the "Boston Medical and Surgical Journal," the treatment of which, involved the removal of the superior maxillary bone, given by that justly distinguished and eminent surgeon, J. C. Warren, M. D. of Boston, and although it is of considerable length, the method of procedure is so minutely detailed, that it furnishes a more correct description of the operation, than any which could otherwise be given.

Case XXVIII.—"The patient, Mr. J. G." says Dr. Warren, "is 35 years old, well constituted, and in every particular strong and healthful, with the exception of the disease which called for this operation. About nine months since he began to be affected with frequent and considerable bleedings from the nose. These bleedings occurred about once a week, and were sometimes profuse. During the occurrence of one of these attacks, he was led to pass the finger deep into the left nostril, and discovered there a tumour about the size of a pea, in the outer side or wall of the cavity.

The bleedings continued and the tumour grew, till it made a visible appearance in the aperture of the left nostril. Alarmed at this, he consulted Dr. Winslow Lewis, who, suspecting a formidable disease, advised him to apply at the Massachusetts General

* Vide Journal de Chirurgie, tom. 1; Parissien Chirurgical Journal, tom. 1.; Œuvres Chir. de Desault, par Bichat, tom. 2.; New London Med. Jour. vol. 1.; Eichorn. Diss. de Polypis in Antro. Highmori. Trans. of a Society for the Improvement of Med. and Chir. Knowledge. Recueil Periodique de la Soc. de Med. tom. 2.; No. 9. Edinburg Med. and Chir. Jour. Nos. 83 and 84; Traite des Maladies Chirurgicales, tom. 6.; Traite des Maladies des Fosses Nazales, New York Jour. of Med. and Surgery, Western Lancet. Cooper's Surgical Dictionary, Benj. Bell's Surgery, vol 4, &c. &c.

Hospital for advice and assistance. He was there examined by Dr. Hayward and myself, and presented the following appearances. The left nostril was filled by a tumour of a deep red colour and soft consistence, discharging blood freely on being subject to a slight touch. A probe could be introduced into the cavity on the inner side of the tumour along the septum of the nose; but, on the outer wall, was soon arrested in its progress by the tumour, which appeared to be connected with this part, and bled so copiously as to prevent a continuance of the examination in this direction. The external appearance of the face being examined, the nose was seen to be tumefied on the left side by the protrusion of the nasal process of the upper jaw, and also by that part of the bone forming the exterior wall of the nasal cavity. On opening the mouth, the hard palate was seen to be the seat of a tumour of an elastic character, oval form, and size sufficient to occupy a considerable portion of this cavity, obviously produced by the pressure of a substance in the nostril above. The mucous membrane of the mouth was not altered in colour or consistence.

On passing the finger through the mouth into the posterior opening of the nostril, this aperture was found to be filled by a soft elastic tumour, similar to that which occupied the anterior aperture. The septum of the nose was slightly inclined into the right nostril.

Such were the history and appearances of this tumour. Its vivid red colour, soft consistence, disposition to bleed, rapid growth, and consequent breaking down of the bones which surrounded it, satisfied me that it was a cephaloma, a malignant fungus, which would destroy the patient's life in a short time unless extirpated: and I, therefore, advised him to enter the hospital, and have it removed. The patient agreed to this course, and went home to make his arrangements.

In nine days after, he entered. When I came to examine the tumour again, I found that, during this short period, it had enlarged considerably; and especially that it had extended to the right side of the palate so far as to leave a small space only between it and the teeth of that side. I was now seriously apprehensive that no operation could wholly eradicate the tumour, and felt much doubt whether it would be expedient to attempt one, in itself always severe, and which in this case would he attended with dangerous bleeding. After weighing the arguments on both sides for three or four days, I came to an affirmative conclusion, provided other gentlemen were of the same opinion. On the Saturday following, the 4th of December, a consultation was held, consisting of Drs. Hayward, Townsend, and Holmes, and these gentlemen being satisfied that as there was no other ground of hope for the patient, and that he must die in a most distressing manner, the operation was decided on, and immediately after executed.

The principal difficulties I anticipated in this operation were the following:—1. Profuse bleeding, which the character of the tumour, the tendency of blood to the head produced by it, and the fulness of the patient's habit, seemed to promise. 2. Impracticability of dividing the bones without sawing, as the patient was of an aspect which indicated unusual solidity of the osseous texture. 3. Fatal syncope, from the quantity of blood lost and the pain of the operation.

To obviate these dangers I proposed—1. Compression of the carotid arteries, tying of the wounded vessels when they bleed freely, and the use of the actual cautery. 2. Division of the bones by the cutting forceps, which I had caused to be made and used for the last twenty years. 3. Waiting occasionally to give the patient time to recover, and recruiting him with cordials.

Every thing being arranged, the patient was placed in a chair, his head well supported, and the operation was then begun in presence of the medical class and a considerable number of medical gentlemen of the city.

I made an incision from the middle of the external edge of the left orbit to the left angle of the mouth, down to the bone. A most copious gush of blood succeeded. The internal flap was then quickly dissected up to the middle of the nose, cutting up at the same time the cartilage of the left wing of the nose, and freeing the globe of the eye from the inferior part of the socket by the division of the inferior oblique muscle, the fascia of the eye, and the periosteum. The outer flap was then rapidly dissected from the os malæ and os maxillæ, and around the latter bone as far as its union with the pterygoid process of the sphenoid; but the uniting space was not at this time penetrated on account of the large pterygoid branch of the internal maxillary, which would have been difficult to secure in this stage of the operation.

The two flaps being separated, the anterior extremity of the spheno-maxillary fissure was perforated, and I then proceeded to the division of the bones. The os malæ was attached directly opposite to the perforation in the spheno-maxillary fissure. The cutting forceps were then applied to the broadest part of the malar bone, and divided it smoothly in a few seconds. Second, the same instrument was applied at the internal angle of the eye, in an oblique direction from the lower edge of the orbit to the lower termination of the os nasi. Here the projection of the tumour into the orbit occasioned some dfficulty, from the little space left for its introduction into the orbit; but, the instrument being fixed, the bone was divided without difficulty.

In the meantime, the blood continued to flow in torrents. One considerable artery required immediate ligature: and the bleeding of the others was controlled by compression of the carotid artery. The mouth of the patient filling with blood, frequent pauses were required to afford him an opportunity of ejecting it, and occasionally he was recruited by a little wine.

The most difficult part of the operation remained; that of dividing the sound from the unsound parts within the mouth, and separating the maxillary from the sphenoid and palatine bones without injury to the latter; so as to leave the patient the whole of the soft palate, with the palatine plate of the os palati to support it. In order to accomplish this without dissection, I made an incision through the mucous membrane of the hard palate, beginning at the edge of the palatine plate of the os palati, and extending the incision forwards to the external edge of the jaw, then upwards across the alveoli into the bone. To facilitate this incision, the middle incisor tooth of the left side was taken out in such a way as to break the anterior part of the alveolus. Then by a single stroke of the cutting forceps the upper maxillary bone was divided, and its palatine plate cut through as far as its junction with the os palati. In order to separate the palatine plates of the maxillary and palatine bones, I hoped to be able to clear the mouth of blood for a moment to make a transverse cut between these plates. But to see was impossible, from the flow of blood. Therefore, passing the fore-finger of the left hand into the mouth, I felt the last molar tooth, and turning the pulp of the finger forwards to receive and support the instrument, I struck a strong-pointed knife through the hard palate at the union of the maxillary and palate bones, separated these bones, and was able also to separate the maxillary bone from the pterygoid process of the sphenoid, and thus accomplished the disunion of all the bones concerned. Finally, the knife was passed externally behind the upper maxillary bone into the space between this and the pterygoid process, to divide the second branch of the fifth pair of nerves. This was done by a stroke of the instrument, and the patient made a great cry, evincing that this nerve had been reached.

Seizing the bone with the left hand by its orbitar and alveolar portions, it was by a gradual movement started from its situation, and aided by a few touches of the knife, its remaining periosteal attachments were divided, and the whole bone and tumour dislodged from the face.

The patient having lost much blood, had now become faint, and was, therefore, placed on a table. The portion of swelled mucous membrane on the right side of the palate was cut off with ease, and it now only remained to arrest the hemorrhage. A ligature was applied to the superior ethmoidal branch or continuation of the maxillary artery. The hemorrhage from a second artery also required to be arrested. This was not easily done, for it was impossible to discover the orifice of the wounded vessel. It was, therefore, touched with caustic potass, and lint applied to it. As the bleeding might recur, the wound was not immediately brought together, but was covered with a cold-water compress, and the patient left in the operating theatre. He was able to swallow and speak, notwithstanding his exhaustion and the length of the operation.

The time expended during the operation I do not know, having always considered it the part of folly to measure an operation by time, rather than the exigencies of the case. I was informed, afterwards, it was over forty minutes. The principal part of this time was expended in waiting for the patient to relieve his mouth and throat of blood, which appeared to embarrass him more than

I had expected. But the time employed in the incisions. both of the soft and hard parts, was short, and certainly could not have exceeded ten minutes.

In three hours after the operation, no bleeding having occurred, the wound was dressed by passing five sutures and applying a cloth of four thicknesses wet in cold water, to be moistened from time to time; and then he was carried to his bed. He passed the night rather uneasily; but the next day he was more quiet. The pulse, for four or five days after the operation, varied from 80 to 112; at the end of six days it was 72. The third, day, the wound being wholly united, the stitches were withdrawn by Mr. Hayward, the house-surgeon, at my request. In two or three days, the patient was able to take softened bread; and, in three weeks from the operation, went home to pass the Christmas with his family—in two days after which, he was discharged. At the present time, eight weeks after the operation, he is at home, takes food freely, and speaks intelligibly. The left eye, at first much swelled, is in a natural state, and he uses it without uneasiness. On the left side of the palate there is an aperture of a triangular form. Through this, the os ethmoides may be felt, the projections of which were mistaken by the patient for a return of his disease. The food occasionally passes through this aperture into the nostrils, and embarrasses the patient momentarily. The soft palate is entire. There is a slight paralysis of the left side of the upper lip, from the division of the facial nerve; and a want of sensibility in the left side of the nose and the left upper lip, from the division of the second branch of the fifth pair of nerves.

*Description of the Tumour.*—The tumour, after its removal, exhibited the following appearances. At its summit appeared the lower floor of the orbit of the eye, at the inside of which was a portion of the nasal process of the os maxillary superius. On its outer part projected one half of the os malæ; below appeared the left half of the palate, with the exception of the part which belongs to the palatine plate of the os palati. A portion of the fossa canina, and the whole alveolar margin, with the correspondent teeth, were visible. On the inner wall of the mass appeared three considerable red-coloured lobes, attached to the outer and inferior part of the maxillary cavity, by something like a pedicle, about an inch in diameter—the three lobes being connected at their attachment, but separated at their internal or nasal extremity into an anterior, middle, and posterior lobe. The superior maxillary nerve was seen in and behind the orbit. The whole was covered by membranes which separated it from the parts in contact. One lobe had made its way through the bone of the face; the others through the partition between the nostril and antrum."

Having now quoted the description of the operation and tumour, I shall notice but a few of the remarks that follow. "The texture of the tumour," says Dr. Warren, "was in consistence somewhat spongy and elastic, and was very vascular." Again he observes: "In order to judge of the propriety of operating in such cases, we must distinguish from each other the different tumours which begin in the maxillary cavity and extend into the nostril, and raise the bony parietes of the face, orbit, and palate. I have seen four different species of such tumour. First, the osteo-sarcoma, of the upper maxillary bone; second, the fibrous tumour; third, scirrhoma; and fourth, cephaloma."

"The first, osteo-sarcoma, is the most formidable in appearance, and attains the greatest size. Its growth is rapid and luxuriant; it breaks down the surrounding bones, and produces enormous deformity. This affection, terrible as it is in appearance, is tractable by operation, and its careful removal is generally followed by a successful result. The second, fibrous tumour, is of slower growth, and more limited in its ravages. This may be removed with a reasonable certainty of its not returning. Third, scirrhoma. This form of tumour of the antrum is characterized by its hardness, the pains which attend it, its moderate growth and certain fatality. Fourth, the cephalomatous is rapid in its growth, and of a spongy texture, produces excessive bleedings, and terminates by death unless removed at an early stage."

It was for the removal of this fourth species of tumour that Dr. Warren operated, and although more than a year has elapsed since the operation was performed, there are no indications, so far as I have been able to learn, of a return of the disease.

In remarking upon the causes of malignant tumours, Dr. W. argues that those who hold that such morbid production, "are necessarily and early the result of a contaminated circulating fluid," "must of course believe that every operation for their removal," "is utterly unavailing," and, while he admits "that a great number" "are followed by signs of a general vitiation of the blood," he believes that inasmuch as they are not always reproduced after their extirpation, that they often attack persons who are unaffected with any general or constitutional vice whatever.

I fully concur in the opinion, that, if all malignant tumours resulted from a vitiation of the circulating fluid, independently of all other causes, no operation for their removal would be successful, but I cannot admit that, inasmuch as they are sometimes successful, it follows, that individuals unaffected with any constitutional taint or vice, may, and not unfrequently do, become the subjects of them. I do not think that this conclusion is warranted by the premise, but having already endeavoured to show, that local causes alone, are incapable of producing them, and that they were dependent, both upon these, and some constitutional vice or taint, it will not be necessary to repeat what has before been said upon the subject.

In conclusion, I would remark, that Professor Pattison proposed in 1820, for the dispersion of fungous tumours of the maxillary sinus, the tying of the carotid artery. He was induced to recommend this method of treatment, from the consideration, that the "capability of action of a part, is proportioned to its vascularity," and that thus by cutting off the circulation of blood to it, the morbid growth would slough and be thrown off. He says this practice has been successful where it has been adopted, in all the cases that have come to his knowledge.*

### EXOSTOSIS OF ITS OSSEOUS PARIETES.

The osseous walls of the maxillary sinus sometimes become the seat of bony tumours—a disease designated by medical writers by the name of exostosis. This, however, is not an affection peculiar to the bony parietes of this cavity; all of the osseous structures of the body are liable to be attacked by it.

Exostosis, like many other diseases, presents several varieties. It is divided by some writers into true and false, the one consist-

* Vide Appendix to Surgical Anatomy of the Head and Neck, pages 477–8.

ing of a tumour composed wholly of bone, or nearly so, and the other, of a tumour composed both of ossific matter and fungous flesh, or of a mere thickening of the periosteal tissue.* Sir Astley Cooper divides exostosis into periosteal, medullary, cartilaginous and fungous. The first consists of a deposition of bony matter on "the external surface of a bone and the internal surface of its periosteum," and to both of which it firmly adheres. The second consists of "a similar formation, originating in the medullary membrane and cancellated structure of the bone;" this description of exostosis never attacks the walls of the maxillary sinus. By cartilaginous exostosis he means that which is preceded by the formation of cartilage, which forms the nidus for the ossific deposite." Fungous exostosis he describes to be a tumour not so firm in its consistence as cartilage, but harder than fungous flesh, having interspersed through its substance spicula of bone, of a malignant character, and dependent upon some peculiar constitutional diathesis, and action of vessels.† This species of exostosis differs but little, if at all, from osteo-sarcoma.

Exostoses differ as much in shape as they do in structure. They sometimes rise abruptly from the surfaces of bones by a narrow and circumscribed base, projecting in large irregularly or spherically shaped masses; at other times they rise very gradually, covering a larger surface of the affected bone, but less massy and with limits less perfectly defined. An exostosis has been known to occupy the whole extent of the surface of a bone. "The whole external surface of one of the bones of the skull was found occupied by an exostosis, while the cerebral surface of the same bone was in a natural state."‡ Both sides and the whole thickness of bones are occasionally affected by this disease. But this is what Sir Astley Cooper calls periosteal exostosis.

This disease is said to attack some bones more frequently than others. Those of the skull, the lower jaw, sternum, humerus, radius, ulna, femur, tibia and bones of the carpus are said to be the most subject to it.§ It also, very frequently attacks the upper jaw, and none of the bones of the body, in fact, are exempt from it.

* Vide Dictionnaire des Sciences Medicales, t. xiv. p. 218.

† Surgical Essays, part 1, page 155.

‡ Vide last American edition of Cooper's Surgical Dictionary, p. 362.

§ Vide last Am. ed. of Cooper's Surgical Dictionary, p. 362.

The texture of exostoses is sometimes spongy and cellular, at other times it is very dense. Dr. E. Carmichael, a distinguished surgeon and physician, formerly of Fredericksburg, but now of Richmond, Va., described to me, about four years since, an exostosis of the superior maxillary, which had, a short time before, fallen under his observation, larger than a hen's egg, and as solid as ivory. Exostoses of the roots of the teeth are always hard, exceeding in density, very frequently, even tooth-bone; and instances are sometimes met with of osseous tumours upon other bones possessed of nearly an equal degree of solidity. Exostoses of this description grow less rapidly than those which are more cellular; but they notwithstanding sometimes acquire a very large size: it is not, however, uncommon for such, after having attained a greater or less size, to cease to grow, and "remain stationary" through life, without giving rise to any very serious or unpleasant consequences.*

Exostoses sometimes attain an enormous size, and especially upon cylindrical bones; very large ones too, are frequently met with upon the maxillæ. The largest one however, I believe, of the maxillary sinus, of which medical history furnishes any account, is exhibited upon a specimen of morbid anatomy, presented in 1767, by M. Beaupreau, to the French Academy. A description and drawing of this tumour is contained in the Memoirs of the Royal Academy of Surgery, but we have no account of the history of its formation, nor of the symptoms that resulted from it. The tumour occupies the whole of the right maxillary sinus, and several of the neighbouring bones are involved in it. It is very large near its base and projects from the lower part of the orbit, forward and downward, six inches. Its largest circumference, is said to be one foot. The upper part of the maxillary bone, says Bordenave, projects on the side of the orbit, and straightens the cavity; the *os unguis* is included in the mass of the tumour and is represented as being nearly effaced. The nasal bones of the left side are displaced, and the right nostril entirely closed up, and the exostosis projects so much on the left side as to be nearly underneath the malar bone. The inferior part of the maxillary bone, says our author, is so extended near its base,

* Vide Cooper's Surgical Dictionary, p. 363.

that it inclines obliquely to the left, and the pterygoid apophyses of this side are larger than those of the other. The malar bone is described as being involved in the upper and external part of the exostosis, which extends to the left maxillary bone.

Exteriorly, says Bordenave, the tumour had a smooth and polished appearance, its upper part was very hard, inferiorly its substance had become thinner, was wanting in some places, and the interior of the exostosis was exposed. The substance of the bone here was spongy and serous, and in appearance, not unlike pumice stone. The walls were thick, and measured in some places one inch.*

From this brief description, taken from the one given of it by Bordenave, some idea may be formed of the dimensions and appearance of this enormous and most remarkable exostosis.

A case of exostosis of each antrum is described by Sir Astley Cooper, both of which, forced themselves up into the orbits, and pushed the eyes from their sockets. One of which made its way into the brain, and caused the death of the patient.†

Mr. Thomas Bell does not believe in the occurrence of "true exostosis upon the bony parietes" of this cavity,‡ but too many examples have presented themselves, to leave any room for doubt upon the subject. Although none may never have fallen under his own immediate observation, there are many well authenticated cases on record,—the details of some of which, I shall presently give. But apart from these, I think it would be difficult to assign any sound reasons for supposing that the osseous walls of this cavity should be more exempt from the disease than the other bones of the body.

*Symptoms.*—The attacks of exostoses of the walls of the maxillary sinus, are generally so insiduous, that the presence of the disease, is not for a long time, even suspected. Those which result from venereal vice, Boyer says, are preceded by acute pain, extending at first to almost every part of the affected bone, but which afterwards confines itself to the affected portion. Those which are occasioned by scrofula, the same writer tells us, are

* Vide Memoires de l'Academie Royale de Chirurgie, t. xiii. obs. xii. p. 412.

† Surgical Essays, part 1. p. 157.

‡ Vide Anat. Phys. and Diseases of the Teeth, p. 281.

attended by a duller and less severe pain, and that the symptoms of those resulting from causes purely local, such, for example, as a blow, are very similar.* But these signs are common to the disease wherever it may be situated, and when it is seated in the maxillary sinus, they do not distinguish it from many of the other affections that occur here; for they are often produced by them, as well as by exostoses. And furthermore, the disease, not unfrequently gives rise to other symptoms which are attendant upon several of the other affections of this cavity, so that previously to the distension of its walls by it, it may be confounded with inflammation of the lining membrane, or sarcomatous or other tumours. But after it has filled the sinus, or very considerably thickened its exterior walls, it will cause them to offer a firmer resistance to pressure, than will any of the other diseases of this cavity. When therefore, they have become distended, if they are firm and unyielding to pressure, the presence of exostosis may be inferred.

*Causes.*—There is a difference of opinion among writers on the diseases of the bones, with regard to the causes of exostosis. Certain constitutional diseases, such as "scrofula and lues venerea," are thought by some to give rise to the affection. That the last of these diseases is favourable to its production, is, I believe, admitted by all; but Sir Astley Cooper declares that no evidence has yet been adduced to prove that the former is ever concerned in its production.† Others impute the disease to local irritation produced by contusions, fractures, &c. &c. But it is probably dependent upon both local and constitutional causes, and that neither, independently of the other, is capable of producing it.

*Treatment.*—A variety of plans of treatment have been recommended for this disease, and Bordenave assures us that it may be cured, if suitable remedies are applied before the exostosis has acquired much solidity. Assuming that it sometimes results from constitutional causes, he directs that the treatment should be commenced by the employment of such means as are indicated by the nature of the vice with which the patient may be affected. If a venereal vice be present, the use of mercurial medicines are recommended. The author, last mentioned, says he has known

* Traite des Maladies Chirurgicales, t. iii. p. 545.

† Vide last American edition of Cooper's Surgical Dictionary, p. 363.

it to be successfully treated with mercury.* Topical applications, such as fomentations and cataplasms, have also been found serviceable. Boyer advises poultices of linseed meal, and a decoction of the "leaves of henbane and nightshade." Iodine and mercury have been employed, but not, I believe, with any decided advantage. Sir Astley Cooper thinks the best internal remedy is "oxymuriate of quicksilver, together with the compound decoction of sarsaparilla.† But I believe, with Boyer, that a dispersion of an exostosis can never be effected.‡ Its progress may perhaps be partially arrested, but I do not believe, as many do, that it is ever removed by the absorbents. But it is not advisable to remove exostoses unless they continue to augment and are likely to become dangerous, or are productive of serious inconvenience.

When, therefore, the remedies which have here been mentioned, after having been thoroughly tried, prove unsuccessful, the tumour should be fully exposed; firstly, by the dissection of the gum and other soft parts from the exterior wall of the sinus, and secondly, by the perforation of this cavity with a trephine, or such other instrument as can be most conveniently employed. This part of the operation, though simple, should be conducted with care, but if the tumour be large and attached by a very broad base, its removal will sometimes prove more difficult, yet by means of suitably constructed saws, scissors, knives, &c., it may, in most instances, be easily effected. An external wound through the cheek should always, if possible, be avoided.

The method of operating, however, will be best understood by a description of that pursued in the two following cases. The first was treated by Dr. B. A. Rodrigues, dentist, of Charleston, S. C., and reported by him for the "American Journal of Medical Sciences."

Case XXIX.—"On the 14th of August, 1837, Charity, a servant woman of Mrs. Miller, called on me to ascertain whether I could afford her any relief in her wretched condition. She had been labouring under incessant and agonizing pain in the antrum highmorianum of the right side, which she regarded as the conse-

* Vide Memoires de l'Academie Royale de Chirurgie, t. xiii. p. 403.

† Vide last Am. ed. of Cooper's Surgical Dictionary, p. 365.

‡ Traite des Maladies Chirurgicales, t. iii. pp. 554—557.

quence of the impaired condition of the teeth. On this supposition, she had several of them extracted, without any appreciable abatement of her sufferings. Yet deluded with the belief that some one of the remaining teeth was the secret agent of all she suffered, she persisted in having more extracted. Still the evil continued, the suffering was unabated, the cause undetected; and to add to the depression of her hopes, and the aggravation of her ills, a purulent discharge oozed from the empty sockets of the affected side. She again had recourse to medical advice, hoping that this phasis of her malady might lead to some indication that would relieve her; at least, that it might reveal its hidden sources, its condition, and its prospects of being remediable. And here for the first time, was it suggested that the antrum was in an unsound state.

It was at this moment, under these circumstances, that she applied to me to perform an operation, which her medical adviser declared to be indispensable. At first, I imagined it to be an abscess from the cavity from which the pus was discharged, from the strange sensations experienced, and from the greater frequency of this disease over others peculiar to this part, I inserted a trocar into the socket of the second molar, and, instead of the gush of matter I had expected, the passage of the instrument was intercepted by a hard, dense, impregnable substance. The existence of an exostosis now forced itself on me. To make assurance doubly sure, I had access to several of my medical friends, among whom, was Dr. Geddings. On examination of the part, the consideration of the symptoms, the obstinate nature of the disease, they concurred with me in opinion, that an exostosis was present, and that the sole indication of relief was its extirpation. Accordingly, on the 18th of August, the above gentlemen with several others of the profession were present, when I proceeded to perform the operation. With a common scalpel, I dissected away the gum from the canine tooth to the last molar, raised the flap which it made from the alveolar process, and with a trephine opened into the cavity. Success was easier than had been anticipated in consequence of the carious condition of the process, which was so general on the affected side, as to reach from the second incisor anteriorly to the pterygoid process posteriorly.

In the loss of substance, the external parietes of the cavity shared, so that the bony tumour which filled up and occupied it, could be readily reached. The trephine was applied, the cavity enlarged, and the exostosis removed. It measured in circumference three inches, was light, and cancellated on its surface, but dense and more resisting in its more internal layers. There was little or no hemorrhage to delay the operation, or any application to arrest it. After removing every spiculum of diseased bone, and cleansing out the cavity, the flap was replaced and to nature was entrusted the cure. Granulations sprouted up in full luxuriance, and in the short period of four weeks, the woman was in the enjoyment of excellent health."*

That the foregoing was a case of true exostosis of the maxillary sinus, does not admit of doubt, and it is to be regretted, that more of the early history of the disease and the circumstances connected with its development are not known. They might perhaps lead to a correct explanation of the causes that gave rise to it. The presence of local irritants in the immediate vicinity of this cavity, is proven by the fact that the patient's teeth were in a diseased condition, but to what extent they may have contributed to the production of the exostosis, it is impossible to determine, since we are not furnished with any information concerning the state of her general health. She may have been affected with some constitutional vice, or peculiar habit of body, whereby the osseous structures of the system were predisposed to affections of this description, requiring only the presence of some local irritant to induce the morbid action necessary to their development. That such predisposition did exist, and that such action was excited by the irritation produced by the diseased teeth, I believe would appear, if all the circumstances connected with the previous history of the case could be ascertained.

When the connection of the exostosis is such as to prevent its complete removal, the application of the actual cautery to any remaining portions, will prove serviceable, by causing such parts to be exfoliated. The history of a case is related by M. Bordenave, treated by M. Runge, in which a portion of the exostosis was left, and which ultimately caused the death of the patient.†

* American Journal of Medical Sciences.

† Vide Memoires de l'Academie Royale de Chirurgie, t. xiii. p. 405.

This would probably have been prevented had an exfoliation of the remaining diseased portions of bone, been brought about by an application of the actual cautery.

CASE XXX.*—The subject of this case was a man 33 years of age. He had been for a long time afflicted with a tumour in the region of the right antrum. It depressed the palatine process of the maxillary bone and the palate bone of the affected side in such a manner as to restrict the movements of the tongue, while on the other side it pressed against the floor of the orbit so as to cause a protrusion of the eye. Anteriorly, it had elevated a portion of the maxillary and malar bones which covered it, and extended to the most dependent part of the nose, whilst posteriorly it extended as far as the posterior mouth. Its effects upon the lateral parts were nearly the same as those which it had exerted upon the others.

After having exposed the anterior parietes of the antrum, M. David, sawed from below upwards to the uppermost part of the projection of the tumour, which was of a spherical shape, and nearly three inches in diameter, and after having elevated that part, he discovered the tumour, which was white and hard; although spongy, and bearing a strong resemblance to soft agaric, it occupied the maxillary sinus. It had changed the form of this cavity and increased its dimensions to an extraordinary degree. The greater portion of this hard osseous substance, although firmly adhering to almost every part of its bony envelope, was by a persevering employment of various means, such as the crotchet, elevator, surgeon's rasp, &c. &c., detached by M. David. But in doing this, he inflicted some injury upon the floor of the orbit, and to some portions which still adhered to the palatine process of the maxillary bone, he applied the actual cautery, which was repeated several times.

An opening was formed by this operation four and a half inches deep, and from right to left, of more than three inches, but a cure was notwithstanding speedily effected by it, which, had the use of the cautery been omitted, would not perhaps have been successful.

Exostoses of the maxillary sinus, often give rise to other morbid conditions of this cavity, the remedial indications of which, should

* Vide Memoires de l'Academie Royale de Chirurgie, t. xiii. obs. xi. p 408.

be properly attended to, as should also those of any constitutional affection, vice or habit of body that the patient may be labouring under at the time.

When the exostosis is not complicated with any other disease of the cavity, the restorative energies of nature, after its removal, will generally be all that is required to complete the cure.

### OF WOUNDS OF ITS PARIETES.

The walls of the maxillary sinus are sometimes fractured by blows and pierced by sharp-pointed instruments. Fauchard mentions a case, in which a canine tooth had been driven up into it,* but this is an accident that rarely happens. The instance here alluded to, is, I believe, the only one on record; and as might be supposed, it was followed by severe pain, and it ultimately gave rise to a tumour upon the cheek near the nose, and three fistulous openings, from which a fetid matter was discharged. The sinus however having been opened and the tooth taken from it, a cure was at once effected.

It often happens when the walls of the sinus are fractured from a blow or other mechanical violence, that portions of the bone and foreign bodies are driven into the cavity, and which by remaining there become a constant source of irritation to its lining membrane, and, not unfrequently a hidden cause of other and more malignant forms of disease. Bordenave describes the case of a French officer, who had the walls of one of his maxillary sinuses fractured by a fragment of a bomb. Dressings were applied to the wound, but it did not heal, and upon examination sometime after by M. Allonel, several pieces of bone and a splint which nearly filled the cavity were found. These were removed, but a cure was not immediately effected; a fistulous opening still remained, and it was not until a long time after, when another splinter came away, that the external opening healed.† The same writer mentions the case of a man who had a nail forced head foremost by the discharge of a gun, into his right cheek and maxillary sinus. The opening became fistulous, and although the point of the nail was subsequently discharged, it was not, until

* Le Chirurgien Dentiste, tom. i. page 391.

† Vide Memoires de l'Academie Royale de Chirurgie, t. xiii. obs. xiv. p. 418.

M. Faubert had removed the remaining part, that the fistula closed.*

Wounds of the antrum are almost always complicated with fractures of its osseous parietes, so that the effects resulting from them are more to be dreaded than those which would be produced simply by the penetration of it with a sharp instrument.

*Treatment.*—The nature and extent of the injury inflicted, should determine the treatment most proper to be adopted for wounds of this cavity. Complicated, as they in most instances are, with the presence of extraneous substances in the sinus, the removal of these constitutes the first, and not unfrequently, the only remedial indication. This should never be neglected. When any extraneous bodies, or portions of bone, have been forced into the sinus, these should first be all carefully removed. The external wound should next be dressed with adhesive slips so as to prevent the formation of an unsightly cicatrix. If constitutional symptoms supervene, they should be met with appropriate remedies.

The following interesting case of a wound of the maxillary sinus, inflicted with a dirk-knife, reported by R. S. Welden, student of medicine, and treated by W. H. Donne, M. D., of Louisville, Ky., is taken from the Western Journal of Medicine and Surgery.

CASE XXXI.—"Schuti, a gardener, aged 42 years, a native of Germany, in a rencontre with an athletic man, on the 3d of May, 1840, was struck with a dirk-knife, which entered about an inch above the right superciliary arch, passed through the corresponding eyelid downwards and backwards, evacuating the humours of the eye, and penetrated the antrum. The globe of the eye was divided by a vertical incision, through which the aqueous humour escaped; the iris was extensively detached at the ciliary margin, and could be partially seen through the transparent cornea—its surface being somewhat obscured by small coagula. The hemorrhage was slight and easily controlled by moderate pressure. The patient complained of intense pain in the temple and cheek of the wounded side, shooting deep into the orbit. Three points of interrupted suture were used to approximate the edges of the

* Vide Memoires de l'Academie Royale de Chirurgie, t. xiii. obs. xiv. p. 418.

divided eye. Lint, saturated with laudanum and warm water, constituted the dressing.

May 4th.—Some tumefaction in the eyelid; pulse 110; tongue coated and dry; skin hot; patient has spent a very restless night. Ordered following medicine, tart. emetic, gr. i.; sulph. magnesia, ℥ ss.; to be dissolved in one-half pint of water, and a tablespoonful to be taken every half-hour, until nausea is induced—after which the interval may be increased.

May 5th.—Bowels freely evacuated; pain less; skin moist; pulse 90 and soft. From this period until the wound healed—the space of three weeks—no constitutional symptoms of an untoward character occurred. The patient, however, contended that a portion of the knife-blade remained in the roof of his mouth. But on the most careful examination no foreign body could be detected.

On the 10th of August, 1842, Mr. Schuti called and requested Dr. Donne to examine his mouth, stating that for six months past he had been annoyed by a rough, projecting substance, which some person had informed him was a piece of dead bone, but which he believed to be the point of the knife, that had been driven down into the bone by the violence of the blow. On looking into the mouth, a small black speck was discernible about one-half inch from the interval between the first and second molar teeth. The parts adjacent were somewhat tumefied and inflamed. Dr. Donne made several attempts to extract this body with a pair of common dissecting forceps, but found it immovably fixed in the substance of the bone. By dissecting around it with a bistoury, down to the palate process of the superior maxillary bone, he was enabled to get a firmer hold, and, with a pair of curved tooth-forceps, succeeded in removing a fragment of the blade, one and one-fourth inches in length, and three-fourths in width at the widest part; the extraction was not effected without considerable violence, and was attended with extreme suffering. The fragment came out with an audible snap, which induced those present to suppose, at first, that it had been broken; but on inspecting its surfaces closely, they were found similarly oxidized, and wanting the lustre which a recent fracture had presented. Upon probing the aperture through which the fragment had been extracted, no other piece could be detected. This opening would scarcely admit the curved probe which Dr. Donne passed into the antrum, in order to satisfy himself that the whole of the foreign body was removed. The next day there was a slight discharge from the aperture, though the patient has suffered very little pain since the operation."

The foregoing is certainly one of the most singular cases of which we have any account, and the most remarkable circumstance connected with it is, that no more injury should have resulted from the presence, for so long a time, in the maxillary sinus, of the portion of the blade of the knife that had been broken off in it. In the cases previously noticed, as reported by Bordenave, disease of the mucous membrane of the antrum, and the discharge of a fetid sanies resulted from the presence of the foreign bodies in this cavity. The same effects were also produced in the case described by Fouchard, of the canine tooth which had been forced up into it.

That foreign bodies are sometimes admitted into the maxillary sinus through wounds penetrating its exterior parietes, has already been shown, but that they should gain access to it in any other way, would seem almost impossible. The smallness and peculiar situation of the opening which communicates with it, is such, as one would think would preclude the introduction of extraneous substances of any kind through it, yet they have been found here, when they could not have gained admission in any other way. There are several well authenticated cases on record in which worms have been found in this cavity. The case mentioned by Bordenave in Obs. 12, page 380, vol. 13, of the Memoirs of the Royal Academy, of a diseased maxillary sinus, from which several worms were at different times discharged, does not prove that they obtained admission into it through the nasal opening, and thus, as some writers have conjectured, gave rise to the disease with which it was affected. In this case, a fistulous opening from the cavity, had existed for a long time previously to the discharge of the worms from it, and it is very probable that they introduced themselves through this. A cause sufficient to have produced the disease in the sinus, had been operating for two years, immediately preceding its manifestation. The patient during the whole of this time was affected with pain in the superior teeth of the affected side.

Deschamps says, his colleague of la Charité Hospital, found a worm, in the maxillary sinus of a soldier, whom he was dissecting, four inches long, and the same writer informs us that a similar example is furnished in the Journal of Medicine. The particulars of a case which came under the observation of Mr. Heysham, physician of Carlisle, taken from a work entitled "Medical Commentaries," are contained in Cooper's Surgical Dictionary.* The subject of this case was a strong woman, sixty years of age, who was in the habit of taking a great deal of snuff. She was affected for a number of years with severe pain in the region of the maxillary sinus, which "extended over one side of the head." She was never entirely free from this pain, but it was greater in cold than in warm weather, and for the purpose of obtaining relief she had been twice salivated, and had taken various anodyne medicines. The pain however, instead of being mitigated by these means, became more severe. Her teeth on the affected side were all extracted, and as a last resort the maxillary sinus was perforated. But this, for several days did not give any relief. Injections of bark and "elixir of aloes," were thrown into it, and "on the fifth day a dead insect" of more than an inch in length, and as thick as a "common quill," was removed from this cavity.

But, instances of the introduction of insects or foreign bodies of any description, into the antrum through the nasal opening, fortunately, are so exceedingly rare, that the Memoirs of Medicine do not furnish more than four or five well established examples.

The signs indicative of the presence of insects or foreign bodies in the maxillary sinus, are so obscure, that the fact can only be ascertained by perforating the cavity and on an examination of its interior. Some say that foreign bodies here cause an itching, crawling or tickling sensation in the substance of the cheek. But this is an uncertain diagnosis, for such sensations are not unfrequent in the region of this cavity. That they sometimes cause great pain, is proven by the history of the case related by Mr. Heysham, the particulars of which I have just noticed, but this like the other signs, is not peculiar to occurrences of this sort alone. It is more or less common to all the morbid affections of this cavity.

* Vide Art. Antrum, page 155.

The proper remedial indication for foreign bodies in the antrum, is their removal. When insects are discovered here, injections of oil and tepid water are recommended. This constitutes about all the treatment necessary to be employed in cases of this kind.

### ARTICLE II.

*A Treatise on Mechanical Dentistry.* By Solyman Brown, M. D., D. D. S.

